# Draft Genome Sequence of the Pristinamycin-Producing Strain Streptomyces sp. SW4, Isolated from Soil in Nusa Kambangan, Indonesia

**DOI:** 10.1128/MRA.00912-18

**Published:** 2018-08-23

**Authors:** Saefuddin Aziz, Yvonne Mast, Wolfgang Wohlleben, Harald Gross

**Affiliations:** a Department of Pharmaceutical Biology, Institute of Pharmaceutical Sciences, University of Tübingen, Tübingen, Germany; b Microbiology Department, Biology Faculty, Jenderal Soedirman University, Purwokerto, Indonesia; c Department of Microbiology and Biotechnology, Interfaculty Institute of Microbiology and Infection Medicine (IMIT), University of Tübingen, Tübingen, Germany; d German Centre for Infection Research (DZIF), Tübingen, Germany; University of California, Riverside

## Abstract

Streptomyces sp. strain SW4 exhibited broad-spectrum antibacterial activity toward Gram-positive and Gram-negative pathogens. The 7.5-Mb draft genome sequence gives insight into the complete secondary metabolite production capacity and reveals genes putatively responsible for its antibacterial activity.

## Abstract (precis)

Streptomyces sp. strain SW4 exhibited broad-spectrum antibacterial activity toward Gram-positive and Gram-negative pathogens.

## ANNOUNCEMENT

Indonesia is recognized as a biodiversity hot spot with a high density of macroorganisms and microorganisms (1). Therefore, we hypothesized that Indonesia is a promising source for novel soil bacteria, particularly actinobacterial strains, which are historically known to be prolific antibiotic-producing strains and still represent an important source of chemical diversity and a reservoir to mine for novel chemical structures (2, 3). The discovery of bioactive natural products from Indonesian actinobacteria (4–9) supports this hypothesis.

Accordingly, as part of our ongoing screening program, strain Streptomyces sp. SW4 was isolated from soil of the Indonesian island Nusa Kambangan. It exhibited pronounced antibacterial properties in disk diffusion tests, conducted according to EUCAST guidelines (http://www.eucast.org), particularly toward Escherichia coli, Bacillus subtilis, Streptococcus pneumoniae, and Pseudomonas fluorescens. In order to clarify the active principle for its antibiotic activity and to determine the overall biosynthetic potential of SW4 to produce natural products, we aimed to obtain the whole-genome sequence of this strain.

The strain Streptomyces sp. SW4 was cultivated for 2 days in R5 medium (10) at 30°C and 180 rpm. Genomic DNA of SW4 was extracted and purified using the Genomic-tip 100/G kit (Qiagen). The isolation procedure was carried out following the standard protocol provided by the manufacturer. For cell lysis, 1.0 ml achromopeptidase was added to the cell lysate. Subsequently, a paired-end library was constructed and subjected to sequencing using a PacBio RS II sequencing platform. *De novo* assembly was performed utilizing Hierarchical Genome Assembly Process 3 (HGAP3), whose protocol relies on PreAssembler v1 for filtering, PreAssembler v2 and AssembleUnitig v1 for assembly, BLASR v1 (11) for mapping, and Quiver v1 for consensus polishing using the only unambiguously mapped reads option. HGAP3 settings were kept at the defaults, except for the genome size estimate parameter, which was set to 8.0 Mbp. Overall, 169,628 filtered reads (*N*_50_, 5,290 bp) were assembled to a 7,475,027-nucleotide draft genome at 82.6-fold coverage. The resulting draft genome sequence consists of 4 contigs in total, with a G+C content of 73.3%. The assembled contigs were annotated with the PGAP (12), yielding a total of 6,764 predicted protein-coding sequences. The closest related type strain based on the 16S rRNA gene sequence (1,511 bp) is Streptomyces leeuwenhoekii (LN831790), with 99.0% sequence identity.

A combined manual and automated analysis for secondary metabolism, using antiSMASH 4.0 (13), predicted 18 biosynthetic gene clusters. Seven of these matched known clusters for the antibiotic pristinamycin (14, 15), specifically, the terpenoids hopene, albaflavenone, and isorenieratene (16–18), the siderophore desferrioxamine (19), the pigment melanin (20), and the osmolyte ectoine (21). The production of pristinamycins was experimentally confirmed by liquid chromatography-tandem mass spectrometry (LC-MS/MS) and high-resolution mass spectrometry (HR-MS) analysis (22). The remaining clusters were predicted to encode 1 terpene, 2 siderophores, 1 nonribosomal peptide, 4 polyketides, 1 indole, and 2 bacteriocins.

Thus, the genome sequence of strain SW4 provides the first insights into its antibiotic capabilities and builds the basis for further genome-driven isolation of bioactive natural products from strain SW4.

### Data availability.

This whole-genome sequencing (WGS) project and the partial 16S rRNA gene sequence have been deposited at DDBJ/ENA/GenBank under the accession numbers QKWM00000000 and MH517391, respectively.
